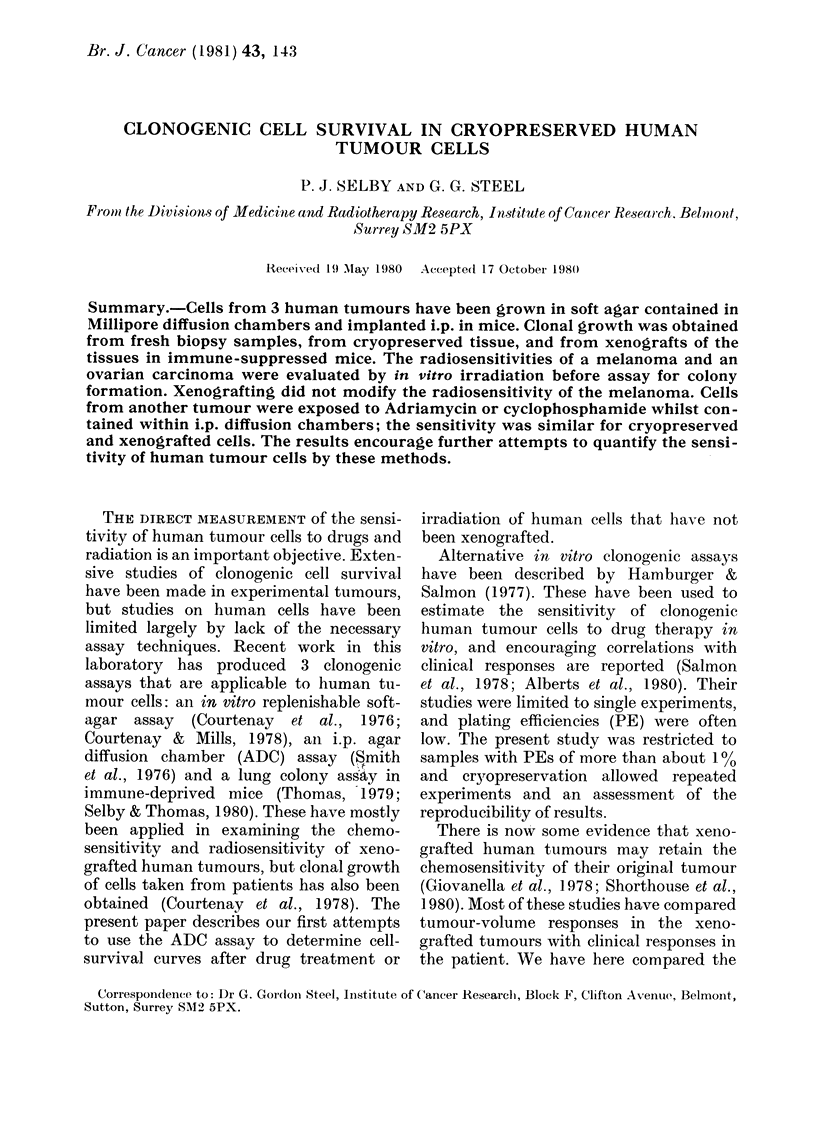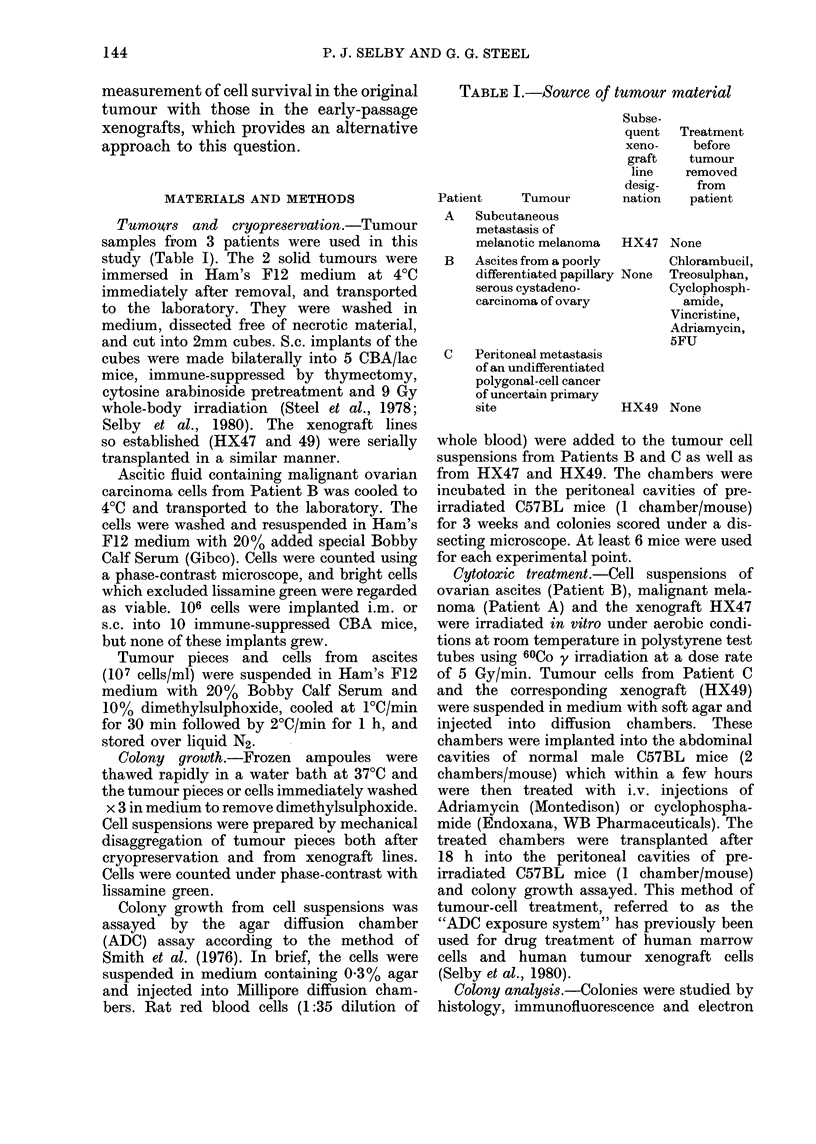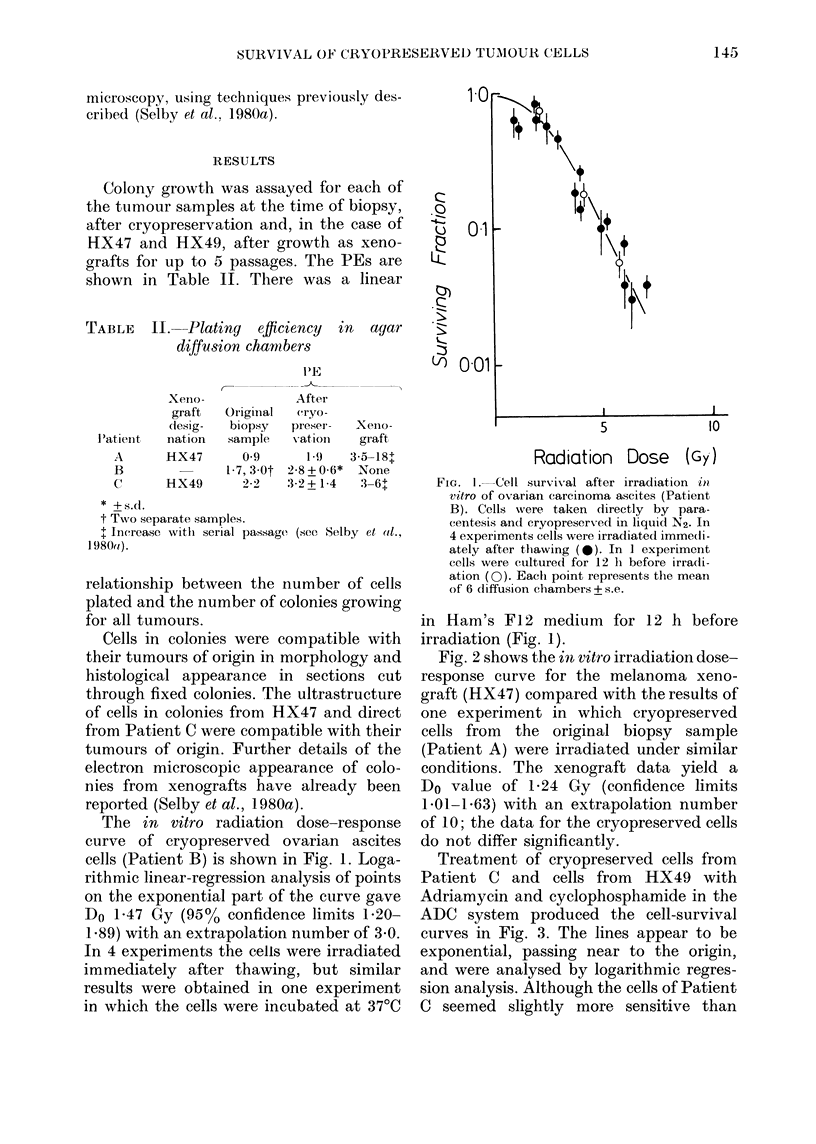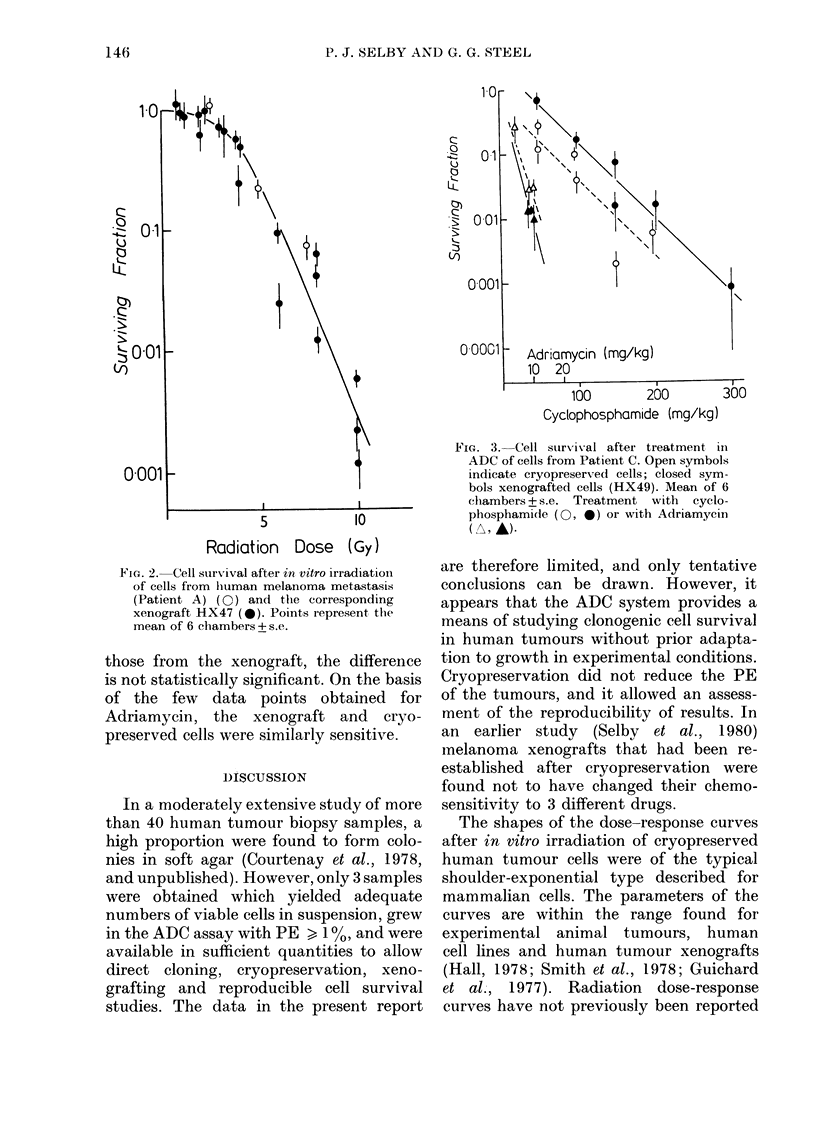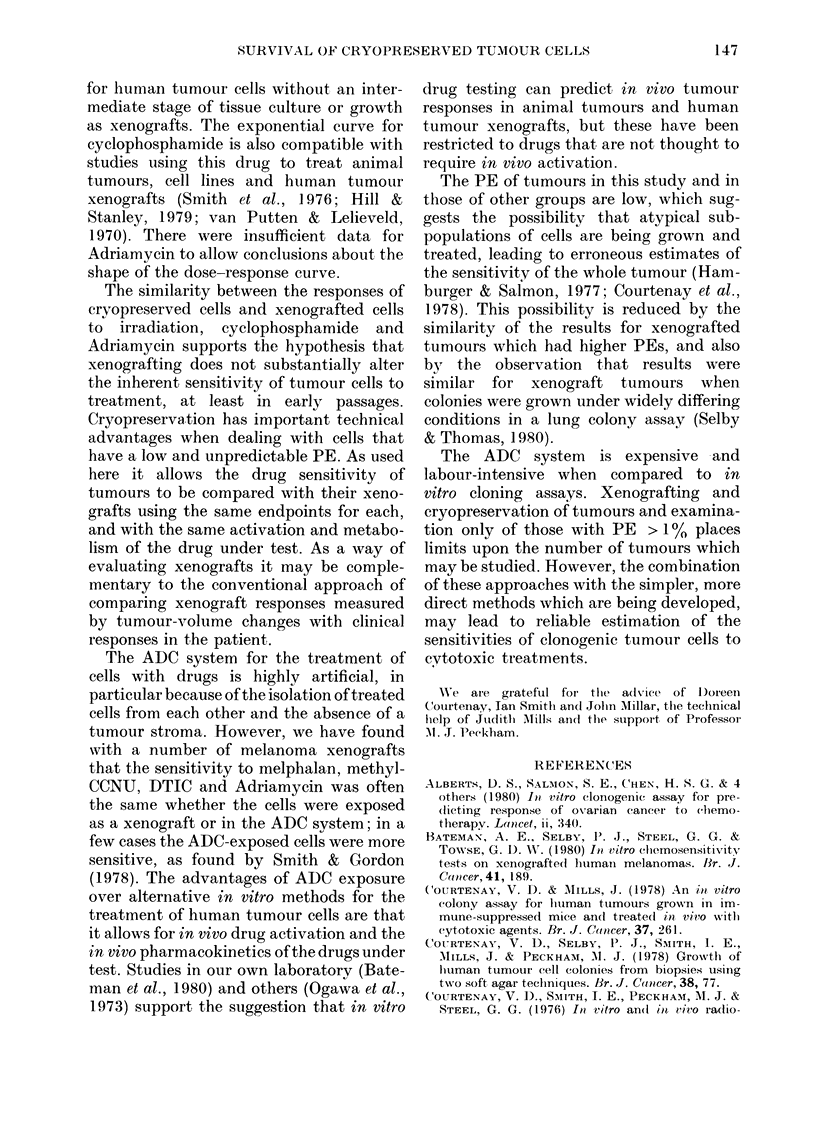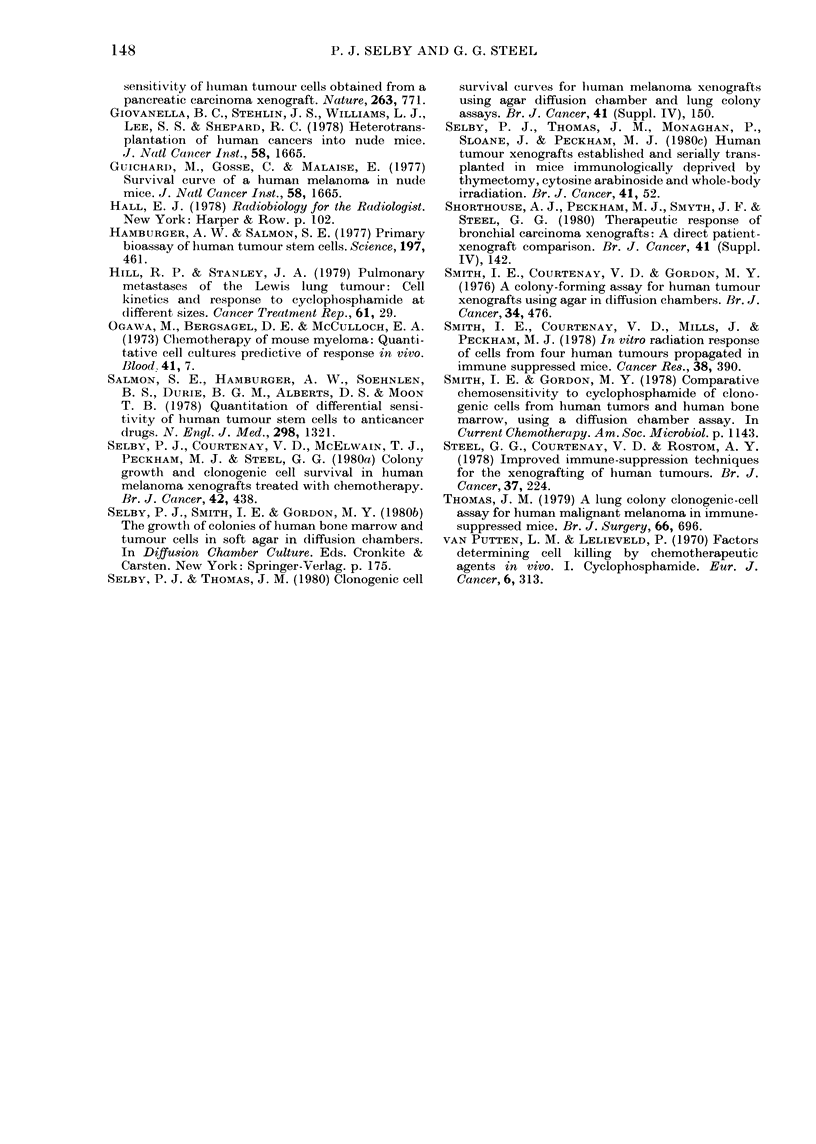# Clonogenic cell survival in cryopreserved human tumour cells.

**DOI:** 10.1038/bjc.1981.22

**Published:** 1981-02

**Authors:** P. J. Selby, G. G. Steel

## Abstract

Cells from 3 human tumours have been grown in soft agar contained in Millipore diffusion chambers and implanted i.p. in mice. Clonal growth was obtained from fresh biopsy samples, from cryopreserved tissue, and from xenografts of the tissues in immune-suppressed mice. The radiosensitivities of a melanoma and an ovarian carcinoma were evaluated by in vitro irradiation before assay for colony formation. Xenografting did not modify the radiosensitivity of the melanoma. Cells from another tumour were exposed to Adriamycin or cyclophosphamide whilst contained within i.p. diffusion chambers; the sensitivity was similar for cryopreserved and xenografted cells. The results encourage further attempts to quantify the sensitivity of human tumour cells by these methods.


*Br. J. Cancer* (1981) **43**, 143

### CLONOGENIC CELL SURVIVAL IN CRYOPRESERVED HUMAN TUMOUR CELLS

P. J. SELBY AND G. G. STEEL

*From the Divisions of Medicine and Radiotherapy Research, Institute of Cancer Research, Belmont, Surrey SM2 5PX*

Received 19 May 1980 Accepted 17 October 1980

**Summary.—Cells from 3 human tumours have been grown in soft agar contained in Millipore diffusion chambers and implanted i.p. in mice. Clonal growth was obtained from fresh biopsy samples, from cryopreserved tissue, and from xenografts of the tissues in immune-suppressed mice. The radiosensitivities of a melanoma and an ovarian carcinoma were evaluated by *in vitro* irradiation before assay for colony formation. Xenografting did not modify the radiosensitivity of the melanoma. Cells from another tumour were exposed to Adriamycin or cyclophosphamide whilst contained within i.p. diffusion chambers; the sensitivity was similar for cryopreserved and xenografted cells. The results encourage further attempts to quantify the sensitivity of human tumour cells by these methods.**

THE DIRECT MEASUREMENT of the sensitivity of human tumour cells to drugs and radiation is an important objective. Extensive studies of clonogenic cell survival have been made in experimental tumours, but studies on human cells have been limited largely by lack of the necessary assay techniques. Recent work in this laboratory has produced 3 clonogenic assays that are applicable to human tumour cells: an *in vitro* replenishable soft-agar assay (Courtenay *et al.*, 1976; Courtenay & Mills, 1978), an i.p. agar diffusion chamber (ADC) assay (Smith *et al.*, 1976) and a lung colony assay in immune-deprived mice (Thomas, 1979; Selby & Thomas, 1980). These have mostly been applied in examining the chemosensitivity and radiosensitivity of xenografted human tumours, but clonal growth of cells taken from patients has also been obtained (Courtenay *et al.*, 1978). The present paper describes our first attempts to use the ADC assay to determine cell-survival curves after drug treatment or irradiation of human cells that have not been xenografted.

Alternative *in vitro* clonogenic assays have been described by Hamburger & Salmon (1977). These have been used to estimate the sensitivity of clonogenic human tumour cells to drug therapy *in vitro*, and encouraging correlations with clinical responses are reported (Salmon *et al.*, 1978; Alberts *et al.*, 1980). Their studies were limited to single experiments, and plating efficiencies (PE) were often low. The present study was restricted to samples with PEs of more than about 1% and cryopreservation allowed repeated experiments and an assessment of the reproducibility of results.

There is now some evidence that xenografted human tumours may retain the chemosensitivity of their original tumour (Giovanella *et al.*, 1978; Shorthouse *et al.*, 1980). Most of these studies have compared tumour-volume responses in the xenografted tumours with clinical responses in the patient. We have here compared the

Correspondence to: Dr G. Gordon Steel, Institute of Cancer Research, Block F, Clifton Avenue, Belmont, Sutton, Surrey SM2 5PX.

measurement of cell survival in the original tumour with those in the early-passage xenografts, which provides an alternative approach to this question.

### MATERIALS AND METHODS

*Tumours and cryopreservation.*—Tumour samples from 3 patients were used in this study (Table I). The 2 solid tumours were immersed in Ham's F12 medium at 4°C immediately after removal, and transported to the laboratory. They were washed in medium, dissected free of necrotic material, and cut into 2mm cubes. S.c. implants of the cubes were made bilaterally into 5 CBA/lac mice, immune-suppressed by thymectomy, cytosine arabinoside pretreatment and 9 Gy whole-body irradiation (Steel *et al.*, 1978; Selby *et al.*, 1980). The xenograft lines so established (HX47 and 49) were serially transplanted in a similar manner.

Ascitic fluid containing malignant ovarian carcinoma cells from Patient B was cooled to 4°C and transported to the laboratory. The cells were washed and resuspended in Ham's F12 medium with 20% added special Bobby Calf Serum (Gibco). Cells were counted using a phase-contrast microscope, and bright cells which excluded lissamine green were regarded as viable. $10^6$ cells were implanted i.m. or s.c. into 10 immune-suppressed CBA mice, but none of these implants grew.

Tumour pieces and cells from ascites ($10^7$ cells/ml) were suspended in Ham's F12 medium with 20% Bobby Calf Serum and 10% dimethylsulphoxide, cooled at 1°C/min for 30 min followed by 2°C/min for 1 h, and stored over liquid $N_2$.

*Colony growth.*—Frozen ampoules were thawed rapidly in a water bath at 37°C and the tumour pieces or cells immediately washed ×3 in medium to remove dimethylsulphoxide. Cell suspensions were prepared by mechanical disaggregation of tumour pieces both after cryopreservation and from xenograft lines. Cells were counted under phase-contrast with lissamine green.

Colony growth from cell suspensions was assayed by the agar diffusion chamber (ADC) assay according to the method of Smith *et al.* (1976). In brief, the cells were suspended in medium containing 0·3% agar and injected into Millipore diffusion chambers. Rat red blood cells (1:35 dilution of whole blood) were added to the tumour cell suspensions from Patients B and C as well as from HX47 and HX49. The chambers were incubated in the peritoneal cavities of pre-irradiated C57BL mice (1 chamber/mouse) for 3 weeks and colonies scored under a dissecting microscope. At least 6 mice were used for each experimental point.

*Cytotoxic treatment.*—Cell suspensions of ovarian ascites (Patient B), malignant melanoma (Patient A) and the xenograft HX47 were irradiated *in vitro* under aerobic conditions at room temperature in polystyrene test tubes using $^{60}Co$ $\gamma$ irradiation at a dose rate of 5 Gy/min. Tumour cells from Patient C and the corresponding xenograft (HX49) were suspended in medium with soft agar and injected into diffusion chambers. These chambers were implanted into the abdominal cavities of normal male C57BL mice (2 chambers/mouse) which within a few hours were then treated with i.v. injections of Adriamycin (Montedison) or cyclophosphamide (Endoxana, WB Pharmaceuticals). The treated chambers were transplanted after 18 h into the peritoneal cavities of pre-irradiated C57BL mice (1 chamber/mouse) and colony growth assayed. This method of tumour-cell treatment, referred to as the "ADC exposure system" has previously been used for drug treatment of human marrow cells and human tumour xenograft cells (Selby *et al.*, 1980).

*Colony analysis.*—Colonies were studied by histology, immunofluorescence and electron

TABLE I.—*Source of tumour material*

| Patient | Tumour | Subsequent xenograft line designation | Treatment before tumour removed from patient |
|---|---|---|---|
| A | Subcutaneous metastasis of melanotic melanoma | HX47 | None |
| B | Ascites from a poorly differentiated papillary serous cystadenocarcinoma of ovary | None | Chlorambucil, Treosulphan, Cyclophosphamide, Vincristine, Adriamycin, 5FU |
| C | Peritoneal metastasis of an undifferentiated polygonal-cell cancer of uncertain primary site | HX49 | None |

microscopy, using techniques previously described (Selby *et al.*, 1980*a*).

### RESULTS

Colony growth was assayed for each of the tumour samples at the time of biopsy, after cryopreservation and, in the case of HX47 and HX49, after growth as xenografts for up to 5 passages. The PEs are shown in Table II. There was a linear relationship between the number of cells plated and the number of colonies growing for all tumours.

TABLE II.—*Plating efficiency in agar diffusion chambers*

| | | PE | | |
|---|---|---|---|---|
| Patient | Xenograft designation | Original biopsy sample | After cryopreservation | Xenograft |
| A | HX47 | 0·9 | 1·9 | 3·5–18‡ |
| B | — | 1·7, 3·0† | 2·8 ± 0·6* | None |
| C | HX49 | 2·2 | 3·2 ± 1·4 | 3–6‡ |

* ± s.d.
† Two separate samples.
‡ Increase with serial passage (see Selby *et al.*, 1980*a*).

Cells in colonies were compatible with their tumours of origin in morphology and histological appearance in sections cut through fixed colonies. The ultrastructure of cells in colonies from HX47 and direct from Patient C were compatible with their tumours of origin. Further details of the electron microscopic appearance of colonies from xenografts have already been reported (Selby *et al.*, 1980*a*).

The *in vitro* radiation dose–response curve of cryopreserved ovarian ascites cells (Patient B) is shown in Fig. 1. Logarithmic linear-regression analysis of points on the exponential part of the curve gave $D_0$ 1·47 Gy (95% confidence limits 1·20–1·89) with an extrapolation number of 3·0. In 4 experiments the cells were irradiated immediately after thawing, but similar results were obtained in one experiment in which the cells were incubated at 37°C in Ham's F12 medium for 12 h before irradiation (Fig. 1).

FIG. 1.—Cell survival after irradiation *in vitro* of ovarian carcinoma ascites (Patient B). Cells were taken directly by paracentesis and cryopreserved in liquid $N_2$. In 4 experiments cells were irradiated immediately after thawing (●). In 1 experiment cells were cultured for 12 h before irradiation (○). Each point represents the mean of 6 diffusion chambers ± s.e.

Fig. 2 shows the *in vitro* irradiation dose–response curve for the melanoma xenograft (HX47) compared with the results of one experiment in which cryopreserved cells from the original biopsy sample (Patient A) were irradiated under similar conditions. The xenograft data yield a $D_0$ value of 1·24 Gy (confidence limits 1·01–1·63) with an extrapolation number of 10; the data for the cryopreserved cells do not differ significantly.

Treatment of cryopreserved cells from Patient C and cells from HX49 with Adriamycin and cyclophosphamide in the ADC system produced the cell-survival curves in Fig. 3. The lines appear to be exponential, passing near to the origin, and were analysed by logarithmic regression analysis. Although the cells of Patient C seemed slightly more sensitive than

FIG. 2.—Cell survival after *in vitro* irradiation of cells from human melanoma metastasis (Patient A) (○) and the corresponding xenograft HX47 (●). Points represent the mean of 6 chambers ± s.e.

FIG. 3.—Cell survival after treatment in ADC of cells from Patient C. Open symbols indicate cryopreserved cells; closed symbols xenografted cells (HX49). Mean of 6 chambers ± s.e. Treatment with cyclophosphamide (○, ●) or with Adriamycin (△, ▲).

those from the xenograft, the difference is not statistically significant. On the basis of the few data points obtained for Adriamycin, the xenograft and cryopreserved cells were similarly sensitive.

### DISCUSSION

In a moderately extensive study of more than 40 human tumour biopsy samples, a high proportion were found to form colonies in soft agar (Courtenay *et al.*, 1978, and unpublished). However, only 3 samples were obtained which yielded adequate numbers of viable cells in suspension, grew in the ADC assay with PE $\geqslant 1\%$, and were available in sufficient quantities to allow direct cloning, cryopreservation, xenografting and reproducible cell survival studies. The data in the present report are therefore limited, and only tentative conclusions can be drawn. However, it appears that the ADC system provides a means of studying clonogenic cell survival in human tumours without prior adaptation to growth in experimental conditions. Cryopreservation did not reduce the PE of the tumours, and it allowed an assessment of the reproducibility of results. In an earlier study (Selby *et al.*, 1980) melanoma xenografts that had been re-established after cryopreservation were found not to have changed their chemosensitivity to 3 different drugs.

The shapes of the dose–response curves after *in vitro* irradiation of cryopreserved human tumour cells were of the typical shoulder-exponential type described for mammalian cells. The parameters of the curves are within the range found for experimental animal tumours, human cell lines and human tumour xenografts (Hall, 1978; Smith *et al.*, 1978; Guichard *et al.*, 1977). Radiation dose-response curves have not previously been reported for human tumour cells without an intermediate stage of tissue culture or growth as xenografts. The exponential curve for cyclophosphamide is also compatible with studies using this drug to treat animal tumours, cell lines and human tumour xenografts (Smith *et al.*, 1976; Hill & Stanley, 1979; van Putten & Lelieveld, 1970). There were insufficient data for Adriamycin to allow conclusions about the shape of the dose–response curve.

The similarity between the responses of cryopreserved cells and xenografted cells to irradiation, cyclophosphamide and Adriamycin supports the hypothesis that xenografting does not substantially alter the inherent sensitivity of tumour cells to treatment, at least in early passages. Cryopreservation has important technical advantages when dealing with cells that have a low and unpredictable PE. As used here it allows the drug sensitivity of tumours to be compared with their xenografts using the same endpoints for each, and with the same activation and metabolism of the drug under test. As a way of evaluating xenografts it may be complementary to the conventional approach of comparing xenograft responses measured by tumour-volume changes with clinical responses in the patient.

The ADC system for the treatment of cells with drugs is highly artificial, in particular because of the isolation of treated cells from each other and the absence of a tumour stroma. However, we have found with a number of melanoma xenografts that the sensitivity to melphalan, methyl-CCNU, DTIC and Adriamycin was often the same whether the cells were exposed as a xenograft or in the ADC system; in a few cases the ADC-exposed cells were more sensitive, as found by Smith & Gordon (1978). The advantages of ADC exposure over alternative *in vitro* methods for the treatment of human tumour cells are that it allows for *in vivo* drug activation and the *in vivo* pharmacokinetics of the drugs under test. Studies in our own laboratory (Bateman *et al.*, 1980) and others (Ogawa *et al.*, 1973) support the suggestion that *in vitro* drug testing can predict *in vivo* tumour responses in animal tumours and human tumour xenografts, but these have been restricted to drugs that are not thought to require *in vivo* activation.

The PE of tumours in this study and in those of other groups are low, which suggests the possibility that atypical subpopulations of cells are being grown and treated, leading to erroneous estimates of the sensitivity of the whole tumour (Hamburger & Salmon, 1977; Courtenay *et al.*, 1978). This possibility is reduced by the similarity of the results for xenografted tumours which had higher PEs, and also by the observation that results were similar for xenograft tumours when colonies were grown under widely differing conditions in a lung colony assay (Selby & Thomas, 1980).

The ADC system is expensive and labour-intensive when compared to *in vitro* cloning assays. Xenografting and cryopreservation of tumours and examination only of those with PE $>1\%$ places limits upon the number of tumours which may be studied. However, the combination of these approaches with the simpler, more direct methods which are being developed, may lead to reliable estimation of the sensitivities of clonogenic tumour cells to cytotoxic treatments.

We are grateful for the advice of Doreen Courtenay, Ian Smith and John Millar, the technical help of Judith Mills and the support of Professor M. J. Peckham.

### REFERENCES

Alberts, D. S., Salmon, S. E., Chen, H. S. G. & 4 others (1980) *In vitro* clonogenic assay for predicting response of ovarian cancer to chemotherapy. *Lancet*, ii, 340.

Bateman, A. E., Selby, P. J., Steel, G. G. & Towse, G. D. W. (1980) *In vitro* chemosensitivity tests on xenografted human melanomas. *Br. J. Cancer*, **41**, 189.

Courtenay, V. D. & Mills, J. (1978) An *in vitro* colony assay for human tumours grown in immune-suppressed mice and treated *in vivo* with cytotoxic agents. *Br. J. Cancer*, **37**, 261.

Courtenay, V. D., Selby, P. J., Smith, I. E., Mills, J. & Peckham, M. J. (1978) Growth of human tumour cell colonies from biopsies using two soft agar techniques. *Br. J. Cancer*, **38**, 77.

Courtenay, V. D., Smith, I. E., Peckham, M. J. & Steel, G. G. (1976) *In vitro* and *in vivo* radiosensitivity of human tumour cells obtained from a pancreatic carcinoma xenograft. *Nature*, **263**, 771.

GIOVANELLA, B. C., STEHLIN, J. S., WILLIAMS, L. J., LEE, S. S. & SHEPARD, R. C. (1978) Heterotransplantation of human cancers into nude mice. *J. Natl Cancer Inst.*, **58**, 1665.

GUICHARD, M., GOSSE, C. & MALAISE, E. (1977) Survival curve of a human melanoma in nude mice. *J. Natl Cancer Inst.*, **58**, 1665.

HALL, E. J. (1978) *Radiobiology for the Radiologist*. New York: Harper & Row. p. 102.

HAMBURGER, A. W. & SALMON, S. E. (1977) Primary bioassay of human tumour stem cells. *Science*, **197**, 461.

HILL, R. P. & STANLEY, J. A. (1979) Pulmonary metastases of the Lewis lung tumour: Cell kinetics and response to cyclophosphamide at different sizes. *Cancer Treatment Rep.*, **61**, 29.

OGAWA, M., BERGSAGEL, D. E. & McCULLOCH, E. A. (1973) Chemotherapy of mouse myeloma: Quantitative cell cultures predictive of response *in vivo*. *Blood*, **41**, 7.

SALMON, S. E., HAMBURGER, A. W., SOEHNLEN, B. S., DURIE, B. G. M., ALBERTS, D. S. & MOON T. B. (1978) Quantitation of differential sensitivity of human tumour stem cells to anticancer drugs. *N. Engl. J. Med.*, **298**, 1321.

SELBY, P. J., COURTENAY, V. D., McELWAIN, T. J., PECKHAM, M. J. & STEEL, G. G. (1980*a*) Colony growth and clonogenic cell survival in human melanoma xenografts treated with chemotherapy. *Br. J. Cancer*, **42**, 438.

SELBY, P. J., SMITH, I. E. & GORDON, M. Y. (1980*b*) The growth of colonies of human bone marrow and tumour cells in soft agar in diffusion chambers. In *Diffusion Chamber Culture*. Eds. Cronkite & Carsten. New York: Springer-Verlag. p. 175.

SELBY, P. J. & THOMAS, J. M. (1980) Clonogenic cell survival curves for human melanoma xenografts using agar diffusion chamber and lung colony assays. *Br. J. Cancer*, **41** (Suppl. IV), 150.

SELBY, P. J., THOMAS, J. M., MONAGHAN, P., SLOANE, J. & PECKHAM, M. J. (1980*c*) Human tumour xenografts established and serially transplanted in mice immunologically deprived by thymectomy, cytosine arabinoside and whole-body irradiation. *Br. J. Cancer*, **41**, 52.

SHORTHOUSE, A. J., PECKHAM, M. J., SMYTH, J. F. & STEEL, G. G. (1980) Therapeutic response of bronchial carcinoma xenografts: A direct patient-xenograft comparison. *Br. J. Cancer*, **41** (Suppl. IV), 142.

SMITH, I. E., COURTENAY, V. D. & GORDON, M. Y. (1976) A colony-forming assay for human tumour xenografts using agar in diffusion chambers. *Br. J. Cancer*, **34**, 476.

SMITH, I. E., COURTENAY, V. D., MILLS, J. & PECKHAM, M. J. (1978) *In vitro* radiation response of cells from four human tumours propagated in immune suppressed mice. *Cancer Res.*, **38**, 390.

SMITH, I. E. & GORDON, M. Y. (1978) Comparative chemosensitivity to cyclophosphamide of clonogenic cells from human tumors and human bone marrow, using a diffusion chamber assay. In *Current Chemotherapy. Am. Soc. Microbiol.* p. 1143.

STEEL, G. G., COURTENAY, V. D. & ROSTOM, A. Y. (1978) Improved immune-suppression techniques for the xenografting of human tumours. *Br. J. Cancer*, **37**, 224.

THOMAS, J. M. (1979) A lung colony clonogenic-cell assay for human malignant melanoma in immune-suppressed mice. *Br. J. Surgery*, **66**, 696.

VAN PUTTEN, L. M. & LELIEVELD, P. (1970) Factors determining cell killing by chemotherapeutic agents *in vivo*. I. Cyclophosphamide. *Eur. J. Cancer*, **6**, 313.